# Poly[μ_2_-chlorido-(μ_2_-3*H* ^+^-1,3,4-thia­diazo­lium-2-thiol­ato-κ^2^ *S*:*S*)silver(I)]

**DOI:** 10.1107/S1600536809012975

**Published:** 2009-04-10

**Authors:** Jian-Ge Wang, Jian-Hua Qin, Pu-Zhou Hu

**Affiliations:** a College of Chemistry and Chemical Engineering, Luoyang Normal University, Luoyang 471022, People’s Republic of China

## Abstract

In the title compound, [AgCl(C_2_H_2_N_2_S_2_)]_*n*_, the Ag^I^ ion has a distorted tetra­hedral geometry, defined by two S atoms of two symmetry-related 1,3,4-thia­diazo­lium-2-thiol­ate ligands and two chloride ions. The Ag^I^ ions are bridged into a two-dimensional network parallel to the *ab* plane by chloride ions and thia­diazole ligands. In the network, the Ag^I^ ions are separated by 4.0316 (12) Å along the *a* axis and by 4.8822 (13) Å along the *b* axis. N—H⋯Cl hydrogen bonds are observed within the network.

## Related literature

For bond-length data, see: Dinger *et al.* (1998 ▶); Wei *et al.* (2008 ▶).
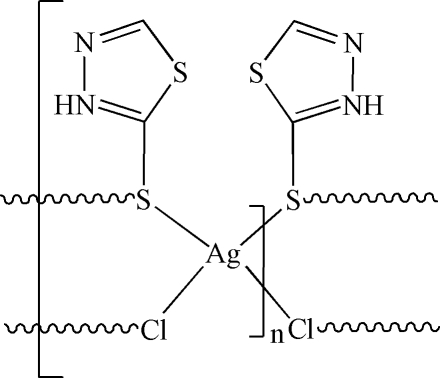


## Experimental

### 

#### Crystal data

[AgCl(C_2_H_2_N_2_S_2_)]
*M* *_r_* = 261.50
Orthorhombic, 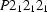

*a* = 4.0316 (9) Å
*b* = 8.473 (2) Å
*c* = 18.368 (4) Å
*V* = 627.4 (2) Å^3^
*Z* = 4
Mo *K*α radiation
μ = 4.19 mm^−1^
*T* = 294 K
0.23 × 0.13 × 0.06 mm

#### Data collection

Bruker SMART CCD area-detector diffractometer
Absorption correction: multi-scan (*SADABS*; Bruker, 1997 ▶) *T*_min_ = 0.446, *T*_max_ = 0.786
4169 measured reflections
1161 independent reflections
1085 reflections with *I* > 2σ(*I*)
*R*_int_ = 0.035

#### Refinement

*R*[*F* ^2^ > 2σ(*F* ^2^)] = 0.031
*wR*(*F* ^2^) = 0.083
*S* = 1.09
1161 reflections
73 parameters
H-atom parameters constrained
Δρ_max_ = 1.22 e Å^−3^
Δρ_min_ = −0.65 e Å^−3^
Absolute structure: Flack (1983 ▶), 438 Friedel pairs
Flack parameter: 0.05 (6)

### 

Data collection: *SMART* (Bruker, 1997 ▶); cell refinement: *SAINT* (Bruker, 1997 ▶); data reduction: *SAINT*; program(s) used to solve structure: *SHELXS97* (Sheldrick, 2008 ▶); program(s) used to refine structure: *SHELXL97* (Sheldrick, 2008 ▶); molecular graphics: *SHELXTL* (Sheldrick, 2008 ▶); software used to prepare material for publication: *SHELXTL*.

## Supplementary Material

Crystal structure: contains datablocks I, global. DOI: 10.1107/S1600536809012975/ci2771sup1.cif

Structure factors: contains datablocks I. DOI: 10.1107/S1600536809012975/ci2771Isup2.hkl

Additional supplementary materials:  crystallographic information; 3D view; checkCIF report

## Figures and Tables

**Table 1:** Selected bond lengths (Å)

Ag1—S2^i^	2.5454 (14)
Ag1—S2	2.5695 (15)
Ag1—Cl1^ii^	2.5897 (15)
Ag1—Cl1	2.6514 (15)

Symmetry codes: (i) 
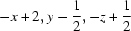
; (ii) 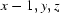.

**Table 2:** Hydrogen-bond geometry (Å, °)

*D*—H⋯*A*	*D*—H	H⋯*A*	*D*⋯*A*	*D*—H⋯*A*
N1—H1⋯Cl1^ii^	0.86	2.38	3.197 (4)	158

Symmetry code: (ii) 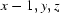.

**Fig. 1.**
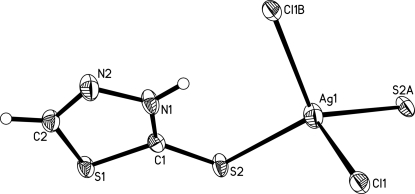
A view of the local coordination of the AgI atom in the title compound. Displacement ellipsoids are drawn at the 30% probability level. Symmetry codes: (A) 2 -x, y - 1/2, 1/2 - z; (B) x - 1, y, z.

**Fig. 2.**
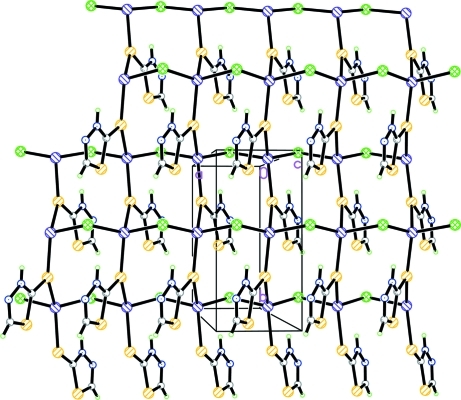
A view of the two-dimensional network parallel to the ab plane.

## supplementary crystallographic information

### Comment

The asymmetric unit of the title compound consists of one Ag^I^ ion, one 1,3,4-thiadiazolium-2-thiolate ligand, and one Cl atom. As depicted in Fig. 1, the Ag^I^ ion is coordinated by two S atoms from two thiadiazole ligands and two Cl atoms in a distorted tetrahedral geometry. The Ag—S and Ag—Cl bond distances (Table 1) are within the range expected for such coordination bonds (Dinger *et al.,* 1998; Wei *et al.,* 2008). The thiadiazole ligand shows a monodentate bridging mode. The adjacent Ag^I^ atoms are bridged by Cl atoms to form chains, which are cross-linked by thiadiazole ligands to form a two-dimensional network parallel to the *ab* plane (Fig. 2). In the network, the Ag atoms are separated by 4.0316 (12) Å along the *a* axis and 4.8822 (13) Å along the *b* axis. Intramolecular N—H···Cl hydrogen bonds are observed in the network (Table 2).

### Experimental

1,3,4-Thiadiazolium-2-thiolate (0.5 mmol) was added at room temperature to a ammonia solution (10 ml) of AgCl (0.5 mmol). After the addition, a colourless precipitate immediately formed and the suspension was stirred for 2 h. The precipitate was filtered off and washed with MeCN. Single crystals suitable for X-ray analysis were obtained by slow diffusion of Et_2_O into a water solution of the solid.

### Refinement

H atoms were positioned geometrically and treated as riding, with N-H = 0.86 Å and *U*_iso_(H) = 1.2*U*_eq_(N), and C-H = 0.93 Å and *U*_iso_(H) = 1.2*U*_eq_(C).

### Crystal data

**Table:**

[AgCl(C_2_H_2_N_2_S_2_)]	*F*(000) = 496
*M**_r_* = 261.50	*D*_x_ = 2.768 Mg m^−^^3^
Orthorhombic, *P*2_1_2_1_2_1_	Mo *K*α radiation, λ = 0.71073 Å
Hall symbol: P 2ac 2ab	Cell parameters from 1870 reflections
*a* = 4.0316 (9) Å	θ = 3.3–27.2°
*b* = 8.473 (2) Å	µ = 4.19 mm^−^^1^
*c* = 18.368 (4) Å	*T* = 294 K
*V* = 627.4 (2) Å^3^	Block, colourless
*Z* = 4	0.23 × 0.13 × 0.06 mm

### Data collection

**Table:**

Bruker SMART CCD area-detector diffractometer	1161 independent reflections
Radiation source: fine-focus sealed tube	1085 reflections with *I* > 2σ(*I*)
graphite	*R*_int_ = 0.035
φ and ω scans	θ_max_ = 25.5°, θ_min_ = 2.7°
Absorption correction: multi-scan (*SADABS*; Bruker, 1997)	*h* = −4→4
*T*_min_ = 0.446, *T*_max_ = 0.786	*k* = −10→10
4169 measured reflections	*l* = −22→22

### Refinement

**Table:**

Refinement on *F*^2^	Secondary atom site location: difference Fourier map
Least-squares matrix: full	Hydrogen site location: inferred from neighbouring sites
*R*[*F*^2^ > 2σ(*F*^2^)] = 0.031	H-atom parameters constrained
*wR*(*F*^2^) = 0.083	*w* = 1/[σ^2^(*F*_o_^2^) + (0.0526*P*)^2^] where *P* = (*F*_o_^2^ + 2*F*_c_^2^)/3
*S* = 1.09	(Δ/σ)_max_ < 0.001
1161 reflections	Δρ_max_ = 1.22 e Å^−^^3^
73 parameters	Δρ_min_ = −0.65 e Å^−^^3^
0 restraints	Absolute structure: Flack (1983), 438 Friedel pairs
Primary atom site location: structure-invariant direct methods	Flack parameter: 0.05 (6)

### Fractional atomic coordinates and isotropic or equivalent isotropic displacement parameters (Å^2^)

**Table:**

	*x*	*y*	*z*	*U*_iso_*/*U*_eq_	
Ag1	0.97702 (12)	−0.03604 (5)	0.18414 (2)	0.04254 (19)	
Cl1	1.4870 (4)	−0.08711 (14)	0.09609 (6)	0.0323 (3)	
S1	0.6628 (4)	0.55205 (15)	0.14333 (7)	0.0346 (3)	
S2	0.9323 (4)	0.25800 (16)	0.21696 (7)	0.0356 (4)	
N1	0.6067 (12)	0.2855 (5)	0.0889 (2)	0.0350 (12)	
H1	0.6188	0.1852	0.0823	0.042*	
N2	0.4580 (15)	0.3800 (5)	0.0387 (2)	0.0420 (13)	
C1	0.7324 (12)	0.3512 (6)	0.1482 (2)	0.0266 (11)	
C2	0.4723 (15)	0.5228 (6)	0.0601 (3)	0.0329 (12)	
H2	0.3863	0.6056	0.0327	0.039*	

### Atomic displacement parameters (Å^2^)

**Table:**

	*U*^11^	*U*^22^	*U*^33^	*U*^12^	*U*^13^	*U*^23^
Ag1	0.0574 (3)	0.0311 (3)	0.0391 (3)	0.0042 (2)	−0.0090 (2)	0.00192 (16)
Cl1	0.0369 (7)	0.0237 (6)	0.0365 (6)	−0.0013 (6)	−0.0016 (6)	−0.0009 (5)
S1	0.0485 (8)	0.0187 (6)	0.0364 (7)	0.0010 (6)	−0.0108 (6)	−0.0037 (6)
S2	0.0546 (9)	0.0215 (6)	0.0307 (7)	0.0065 (6)	−0.0107 (6)	−0.0034 (5)
N1	0.059 (3)	0.019 (2)	0.026 (2)	0.003 (2)	−0.007 (2)	−0.0031 (18)
N2	0.065 (4)	0.027 (3)	0.034 (2)	0.003 (3)	−0.015 (3)	0.0008 (19)
C1	0.032 (3)	0.023 (3)	0.025 (2)	0.000 (2)	0.004 (2)	−0.001 (2)
C2	0.044 (3)	0.026 (3)	0.028 (2)	0.002 (3)	−0.008 (2)	0.001 (2)

### Geometric parameters (Å, °)

**Table:**

Ag1—S2^i^	2.5454 (14)	S2—C1	1.694 (5)
Ag1—S2	2.5695 (15)	S2—Ag1^iv^	2.5453 (14)
Ag1—Cl1^ii^	2.5897 (15)	N1—C1	1.323 (6)
Ag1—Cl1	2.6514 (15)	N1—N2	1.361 (6)
Cl1—Ag1^iii^	2.5897 (15)	N1—H1	0.86
S1—C1	1.727 (5)	N2—C2	1.273 (7)
S1—C2	1.729 (5)	C2—H2	0.93
			
S2^i^—Ag1—S2	120.49 (3)	C1—N1—N2	118.6 (4)
S2^i^—Ag1—Cl1^ii^	116.15 (5)	C1—N1—H1	120.7
S2—Ag1—Cl1^ii^	104.77 (4)	N2—N1—H1	120.7
S2^i^—Ag1—Cl1	102.24 (5)	C2—N2—N1	109.3 (4)
S2—Ag1—Cl1	110.84 (5)	N1—C1—S2	126.8 (4)
Cl1^ii^—Ag1—Cl1	100.56 (5)	N1—C1—S1	108.1 (4)
Ag1^iii^—Cl1—Ag1	100.56 (5)	S2—C1—S1	125.1 (3)
C1—S1—C2	88.6 (2)	N2—C2—S1	115.4 (4)
C1—S2—Ag1^iv^	106.32 (18)	N2—C2—H2	122.3
C1—S2—Ag1	108.09 (18)	S1—C2—H2	122.3
Ag1^iv^—S2—Ag1	145.29 (5)		
			
S2^i^—Ag1—Cl1—Ag1^iii^	−60.05 (5)	N2—N1—C1—S2	−179.1 (4)
S2—Ag1—Cl1—Ag1^iii^	69.61 (5)	N2—N1—C1—S1	0.2 (6)
Cl1^ii^—Ag1—Cl1—Ag1^iii^	180.0	Ag1^iv^—S2—C1—N1	−172.7 (5)
S2^i^—Ag1—S2—C1	−158.63 (19)	Ag1—S2—C1—N1	2.7 (5)
Cl1^ii^—Ag1—S2—C1	−25.46 (19)	Ag1^iv^—S2—C1—S1	8.2 (4)
Cl1—Ag1—S2—C1	82.19 (19)	Ag1—S2—C1—S1	−176.5 (3)
S2^i^—Ag1—S2—Ag1^iv^	13.55 (10)	C2—S1—C1—N1	−0.5 (4)
Cl1^ii^—Ag1—S2—Ag1^iv^	146.72 (12)	C2—S1—C1—S2	178.8 (4)
Cl1—Ag1—S2—Ag1^iv^	−105.63 (12)	N1—N2—C2—S1	−0.9 (7)
C1—N1—N2—C2	0.4 (8)	C1—S1—C2—N2	0.8 (5)

Symmetry codes: (i) −*x*+2, *y*−1/2, −*z*+1/2; (ii) *x*−1, *y*, *z*; (iii) *x*+1, *y*, *z*; (iv) −*x*+2, *y*+1/2, −*z*+1/2.

### Hydrogen-bond geometry (Å, °)

**Table:**

*D*—H···*A*	*D*—H	H···*A*	*D*···*A*	*D*—H···*A*
N1—H1···Cl1^ii^	0.86	2.38	3.197 (4)	158

Symmetry codes: (ii) *x*−1, *y*, *z*.
